# Predicting and Evaluating the Epidemic Trend of Ebola Virus Disease in the 2014-2015 Outbreak and the Effects of Intervention Measures

**DOI:** 10.1371/journal.pone.0152438

**Published:** 2016-04-06

**Authors:** Zuiyuan Guo, Dan Xiao, Dongli Li, Xiuhong Wang, Yayu Wang, Tiecheng Yan, Zhiqi Wang

**Affiliations:** 1 Department of Disease Control, Center for Disease Control and Prevention of Shenyang Military Region, Shenyang, Liaoning Province, China; 2 Department of Epidemiology, Fourth Military Medical University, Xi’an, Shaanxi Province, China; 3 College of Municipal and Environmental Engineering, Shenyang Jianzhu University, Shenyang, Liaoning Province, China; Shanxi University, CHINA

## Abstract

We constructed dynamic Ebola virus disease (EVD) transmission models to predict epidemic trends and evaluate intervention measure efficacy following the 2014 EVD epidemic in West Africa. We estimated the effective vaccination rate for the population, with basic reproduction number (*R*_0_) as the intermediate variable. Periodic EVD fluctuation was analyzed by solving a Jacobian matrix of differential equations based on a SIR (susceptible, infective, and removed) model. A comprehensive compartment model was constructed to fit and predict EVD transmission patterns, and to evaluate the effects of control and prevention measures. Effective EVD vaccination rates were estimated to be 42% (31–50%), 45% (42–48%), and 51% (44–56%) among susceptible individuals in Guinea, Liberia and Sierra Leone, respectively. In the absence of control measures, there would be rapid mortality in these three countries, and an EVD epidemic would be likely recur in 2035, and then again 8~9 years later. Oscillation intervals would shorten and outbreak severity would decrease until the periodicity reached ~5.3 years. Measures that reduced the spread of EVD included: early diagnosis, treatment in isolation, isolating/monitoring close contacts, timely corpse removal, post-recovery condom use, and preventing or quarantining imported cases. EVD may re-emerge within two decades without control and prevention measures. Mass vaccination campaigns and control and prevention measures should be instituted to prevent future EVD epidemics.

## Introduction

The first case of Ebola Virus Disease (EVD) was detected in Guinea in December 2013. Within months, the disease had spread to Liberia, Sierra Leone, Nigeria, and other countries [1, 2]. The World Health Organization (WHO) was notified officially of the rapid evolution of the EVD outbreak on March 23, 2014 [3]. A total of 26,277 EVD cases and 10,884 EVD-related deaths were reported from December 2013 to April 29, 2015 [4]. EVD was transmitted by direct and indirect contact between people. The mean incubation period was 11.4 days, and typical symptoms included fever, nausea, vomiting, diarrhea, and generalized pain [5–7]. The case fatality rate in the 2014 EVD outbreak was approximately 64% among patients who received treatment, and approximately 71% among all known infected patients [3]. The spread of the EVD outbreak was overwhelming and posed a serious threat to public health in Africa due to the high infection rate of EVD, ineffective control measures during the early-onset stage, local funeral customs, and weak epidemic prevention measures [8–12].

Understanding the epidemiological distribution and transmission pattern of EVD during the African outbreak will contribute to improve scientific understanding and to allow the development of more effective control and prevention measures. Other scholars have already analyzed the epidemiological distribution and performance of clinical centers [3, 5, 13], providing valuable information for better understanding the EVD outbreak. However, there is no recognized mathematical model that describes the process of EVD transmission, and that can be used to evaluate the effect of prevention measures. This lack of a model might hinder the process of controlling the disease.

A dynamic transmission model of an infectious disease can be constructed by using the patterns of disease occurrence and transmission, and the relationships between the disease and social factors. Such a model is used to analyze the dynamic characteristics of disease transmission quantitatively, to indicate transmission patterns, and to predict the epidemiological trends of the disease. Dynamic transmission models have been constructed successfully for SARS (severe acute respiratory syndrome), AIDS (acquired immune deficiency syndrome), and malaria to analyze transmission patterns, predict epidemiological trends, and evaluate the effect of intervention measures [14–18]. These models have provided important evidence for the scientific control and prevention of these diseases. In a previous study, researchers constructed an SEIR (susceptible, exposed, infective, removed) model to analyze the 1995 EVD outbreak in the Congo. The constructed model was well fitted and demonstrated to be effective for prediction [19], indicating that a dynamic transmission model can be applied to the analysis of EVD outbreaks. However, the transmission patterns of infectious diseases are affected by many factors and can be variable across different time periods and regions. Therefore, the 1995 outbreak model may not be applicable to the 2014 outbreak, and it is necessary to construct an updated model based on the 2014–2015 transmission patterns and the factors that might influence spread.

Here, we constructed dynamic transmission models for EVD based on the relationships among people in each stage of the disease. The results may aid in predicting epidemiological trends, evaluating the effectiveness of intervention measures, and providing scientific evidence for improving the control and prevention of EVD.

## Results

### Estimation of the effective vaccination rate

The *R*_0_ values and corresponding 95% confidence intervals for EVD in Guinea, Liberia, and Sierra Leone were 1.71 (1.44–2.01), 1.83 (1.72–1.94), and 2.02 (1.79–2.26), respectively [3]. Therefore, at least 42% (31–50%), 45% (42–48%), and 51% (44–56%) of individuals in these respective countries should be immune to EVD.

### Epidemic trends of EVD in the absence of control and prevention measures

**Fig 1:**
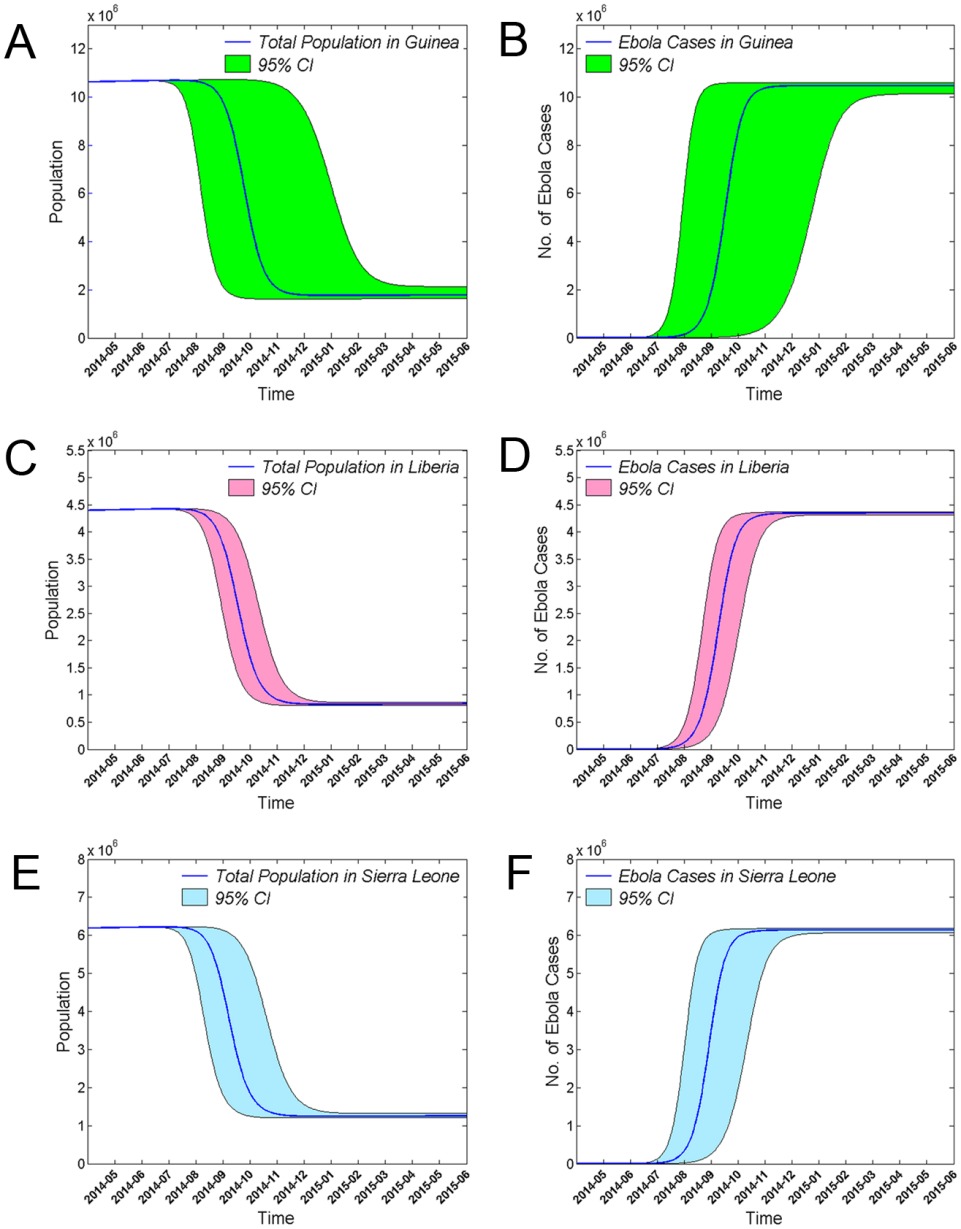
Effects of EVD on total population and EVD case number in the absence of control and prevention measures. Changes in the total number of individuals in the population and in the number of EVD cases in Guinea (A, B), Liberia (C, D), and Sierra Leone (E, F) are shown. The x-axes indicate time in months, and the y-axes indicate population number or the number of EVD cases. The shaded area indicates the 95% confidence interval.

Based on the SIR model, without any outbreak control or prevention measures, the total population number would decrease rapidly in a short period of time (Fig 1). The net growth threshold values for the epidemic in Guinea (0.95), Liberia (1.01), and Sierra Leone (0.90) were all close to 1.0. Therefore, when the parameters of the model are held constant over a sufficiently long time period, the populations of the three countries decrease to constant levels, and the numbers of individuals in the *s*, *i*, and *r* blocks would oscillate with a periodic weakly dampened oscillation, and reach a positive dynamic equilibrium. The equilibrium points for *s*, *i*, and *r* were found to be 58.5%, 0.1%, and 41.1%, respectively, in Guinea, 54.6%, 0.1%, and 45.3%, respectively, in Liberia, and 49.5%, 0.1%, and 50.4%, respectively, in Sierra Leone.

**Fig 2:**
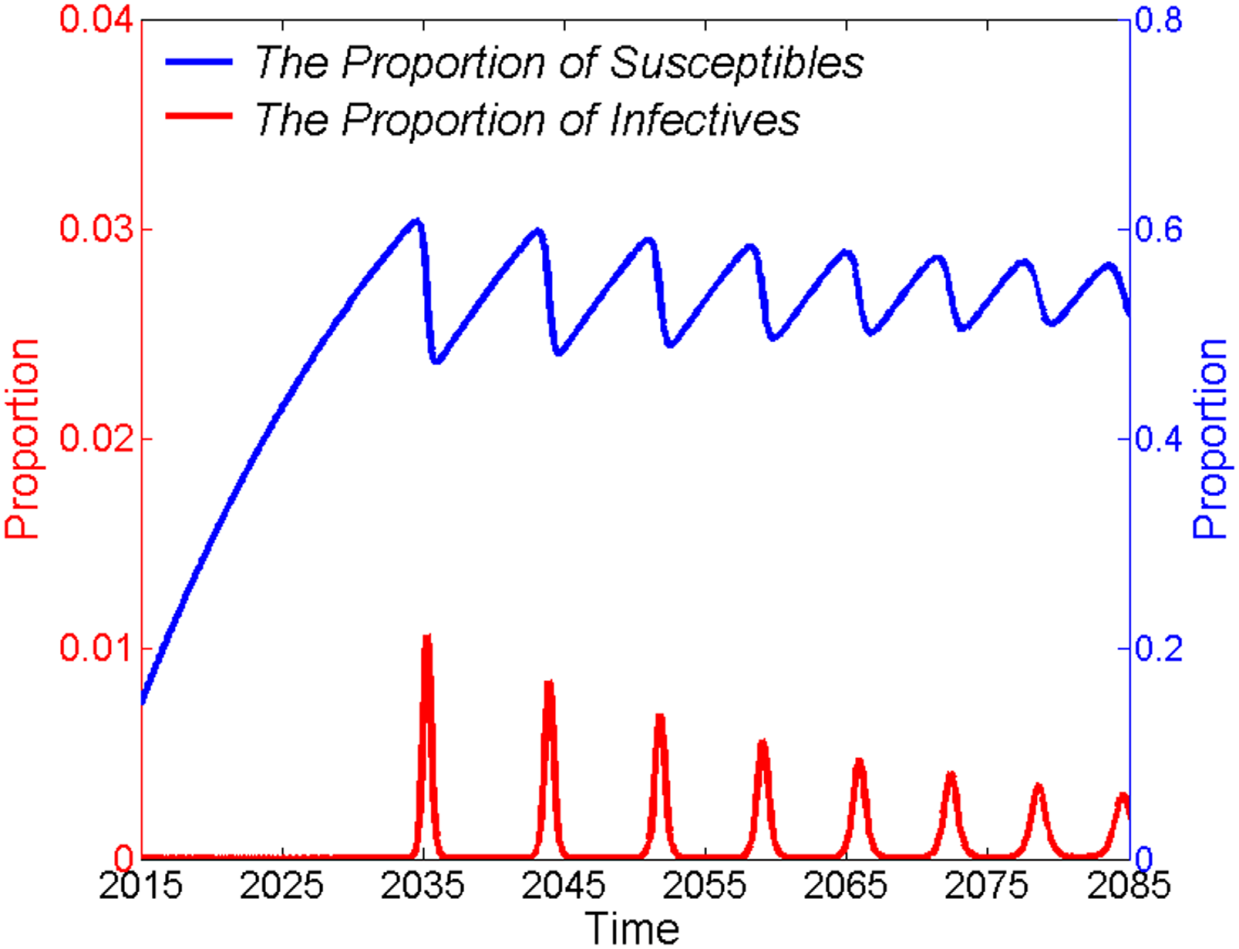
Periodical low dampened oscillation of *s* and *i* around the dynamic equilibrium point. The proportions of EVD cases (left y-axis) and susceptible people (right y-axis), compared to the total population, are shown as functions of time in years. In the absence of control and prevention measures, the *s* and *i* values would oscillate periodically around their dynamic equilibrium points. Meanwhile, periods between oscillations would shorten, and amplitudes would decrease gradually.

Combining the three countries into a single region, the model indicates that if there were no EVD outbreak control or prevention measures in place, EVD would first re-emerge in 2035, and then re-emerge again about 8–9 years later. The oscillation periods would shorten and the amplitudes (severity of EVD epidemic) would decrease gradually until the oscillation occurred with a sustained periodicity of approximately 5.3 years around the positive dynamic equilibrium point (Fig 2).

### Predicting the numbers of EVD cases and deaths

**Fig 3:**
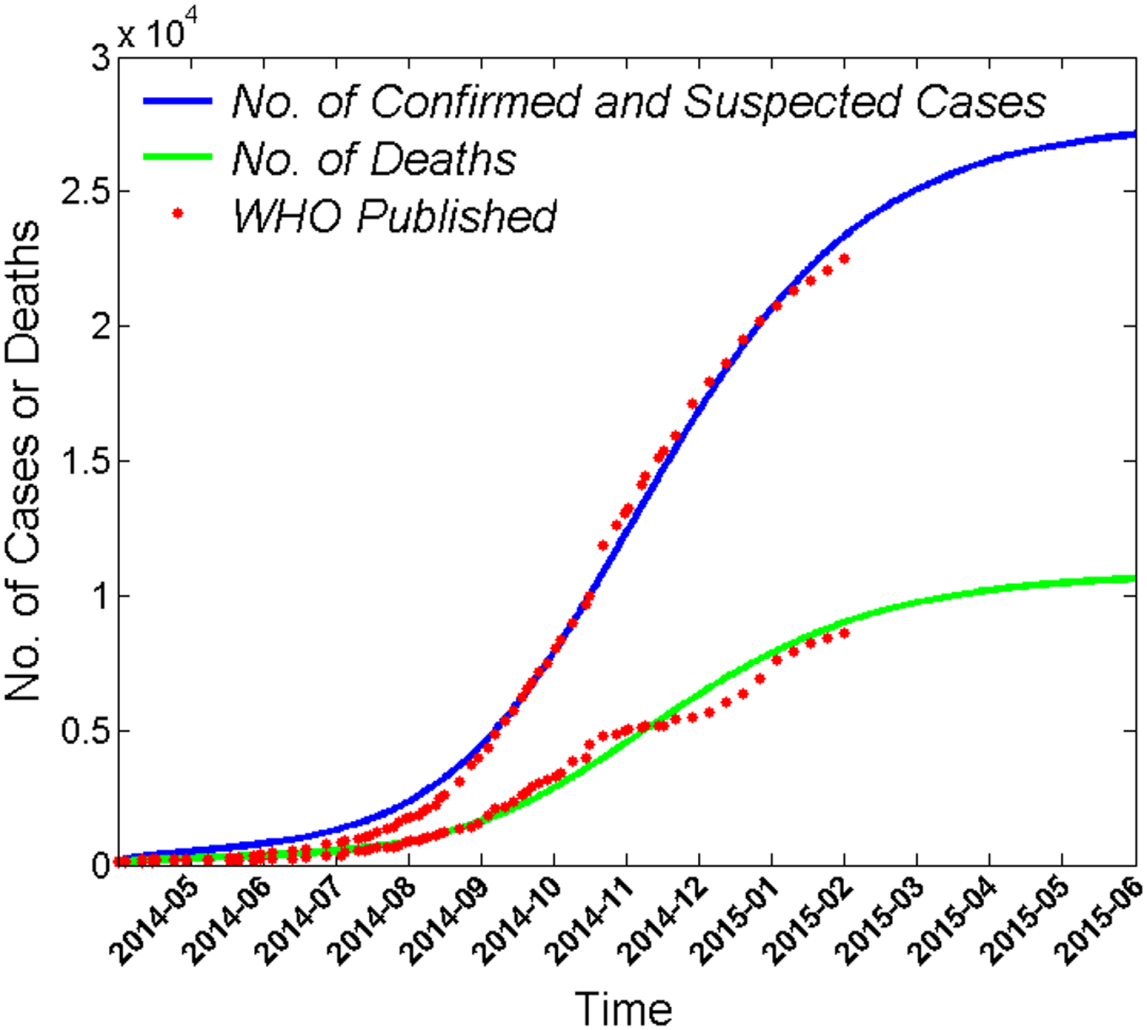
Comparison of the numbers of EVD cases and deaths predicted by the model to those published by the WHO. The cumulative number of EVD cases and deaths are plotted as a function of time *t* in months. The cumulative number of cases predicted curve (blue) includes confirmed and suspected cases. Each red point indicates the number of cumulative cases (blue curve) or deaths due to EVD (green curve) published by the WHO.

The WHO has reported numbers of EVD cases (confirmed and suspected) and deaths due to EVD [20]. These data were used to compare the predicted values from the model to the actual data. The infection rate for patients in an uncontrolled environment (*β*_*I*_), indicates the number of susceptible individuals to be infected by an EVD patient per unit time following a gamma distribution pattern; meanwhile, the *β*_*D*_, *β*_*P*_, and *β*_*R*_ rates reflect an exponential distribution pattern. The numbers of EVD cases and EVD-related deaths predicted by the model were similar to the actual data (Fig 3), indicating that the model simulated the epidemic pattern of EVD accurately.

### Modeling the effects of isolating close contacts on the epidemic trend of EVD

**Fig 4:**
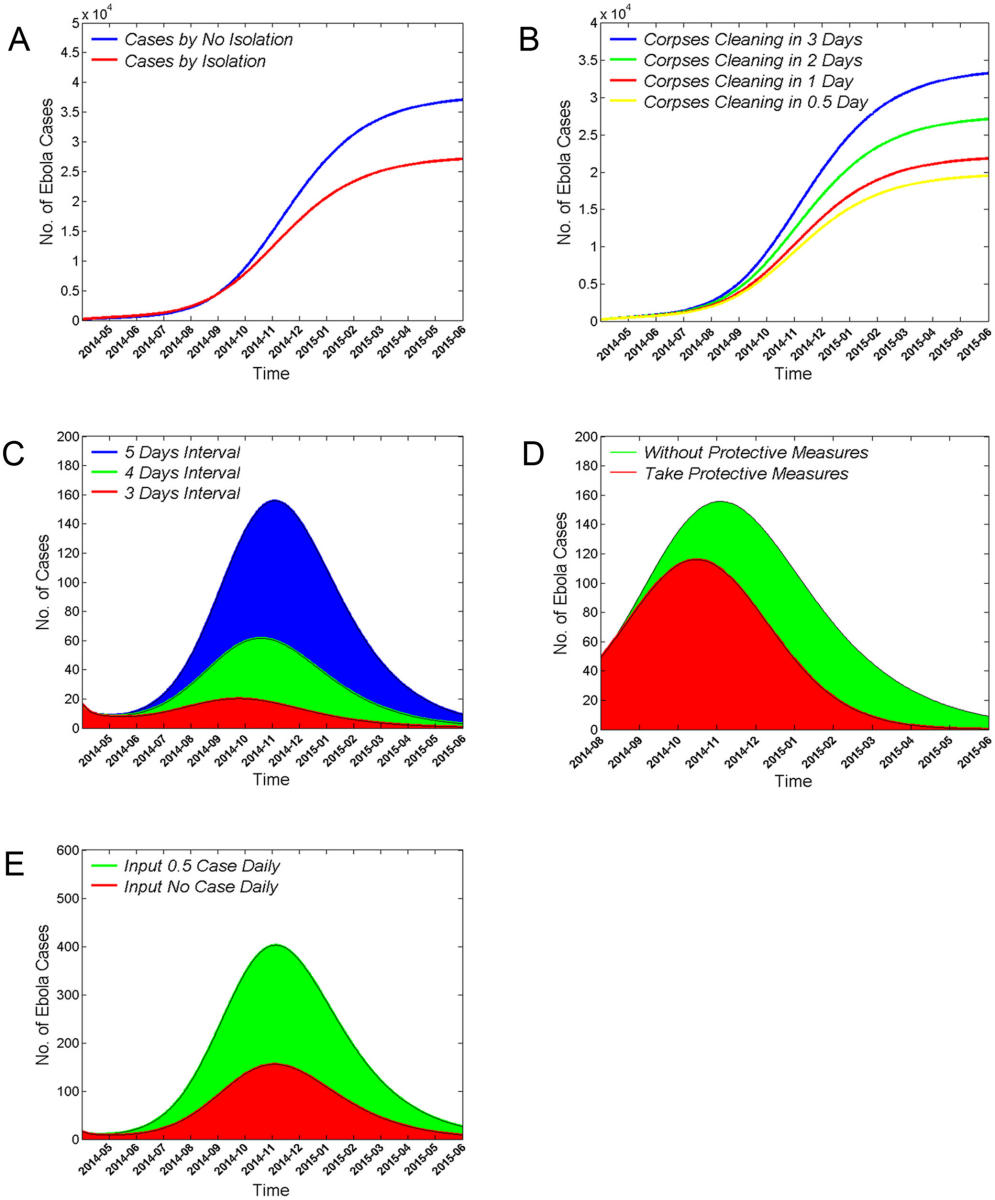
Effects of control and prevention measures on EVD epidemic trends. (A) Summary of the effect of isolating EVD patients’ close contacts on the cumulative number of EVD cases. Note the markedly lower curve with close contact quarantine (red) relative to that without close contact quarantine (blue). (B) Predicted numbers of cumulative EVD cases when corpses of dead EVD patients were allowed to stay in the surrounding environment for 3 (blue), 2 (green), 1 (red), or 0.5 days (yellow). (C) Predicted new EVD cases when the average symptom onset-to-isolation and treatment is 5 days (blue), 4 days (green), and 3 days (red). (D) Predicted reduction in the number of new EVD cases from August 1^st^, 2014 to May 31^st^, 2015 when recovered patients have condom-protected (red) versus unprotected (green) sex. (E) Summary of the effect of cases being imported from other countries on the epidemic trend of EVD. Note that introduction of a new case every other day (0.5 cases/day, green) has a strong effect on amplitude relative to zero case importation (red). In all panels, the number of predicted EVD cases is plotted as a function of time.

Individuals in close contact with EVD patients are a high-risk population and are prone to infecting other susceptible individuals if they are not isolated and closely monitored. Therefore, quarantining the close contacts of EVD patients early is critical to control and prevent transmission of EVD. As shown in Fig 4A, quarantining close contacts of patients decreased the predicted incidence of EVD dramatically. By May 31^st^, 2015, the cumulative number of EVD cases predicted without isolation of close contacts was 37% greater than the predicted number with isolation.

### Effects of decontaminating the corpses of dead EVD patients on the epidemic trend of EVD

Ebola virus is stored in human body fluids. Thus, to prevent the spread of disease, it is critical to take timely measures to decontaminate the corpses of dead EVD patients. Examples of these measures would include burning or deep burial. In contrast, the West African funeral custom of touching the corpses is prone to spreading the disease. The number of cumulative EVD cases could be decreased by shortening the interval between death and corpse decontamination (Fig 4B). Our dynamic model indicates that, reduction of the interval between a person’s death and clearing of the corpse to less than 3 days could have reduced the cumulative number of cases by 19% (2 day interval), 34% (1 day interval), or as much as 41% (0.5 day interval) through May 31^st^, 2015.

### Effects of altering the time from symptom onset to treatment in isolation on the epidemic trend of EVD

The average time from symptom onset to medical treatment in isolation for EVD patients is 5 days [3]. During this time, EVD patients have a high probability of infecting susceptible individuals. Early detection, diagnosis, and isolation can decrease the total number of EVD cases in an open environment, and early treatment can increase the EVD survival rate. A shorter interval between symptom onset and medical treatment in isolation led to fewer EVD cases and an earlier peak in the epidemic curve (Fig 4C). The dynamic model indicates that: if the average interval between symptom onset and treatment were shortened from 5 to 4 days (60% reduction) or 3 days (87% reduction), the number of EVD cases at the peak of the epidemic curve would be markedly decreased in amplitude, and shifted leftward to 16 or 40 days earlier, respectively.

### Effects of recovered EVD patients practicing protected sex on the epidemic trend of EVD

Ebola virus is detectable in the semen of recovered individuals and can be transmitted through sexual contact [21]. Although sexual transmission is not the major route of transmission for EVD, it can still impact the epidemic trend. If recovered patients practiced protected sex using a condom, then the number of EVD cases is reduced and the peak of the epidemic curve is advanced (Fig 4D). The dynamic model indicates that if all recovered individuals had practiced protected sex from August 1^st^ of 2014 onward, then the number of EVD cases at the peak of the epidemic curve would have been reduced by 26% and occurred 19 days earlier.

### Effects of cases imported from other countries on the epidemic trend of EVD

Finally, we analyzed the influence of cases being imported from other countries on the epidemic trend of EVD with the dynamic model. If 0.5 cases were being imported from the uncontrolled outside environment daily, then the number of new EVD cases at the peak of the epidemic curve would be 1.6 times higher than that in the absence of any imported cases (Fig 4E). This finding indicates that imported cases can contribute substantially to EVD epidemic trends.

### Sensitivity analysis

**Fig 5:**
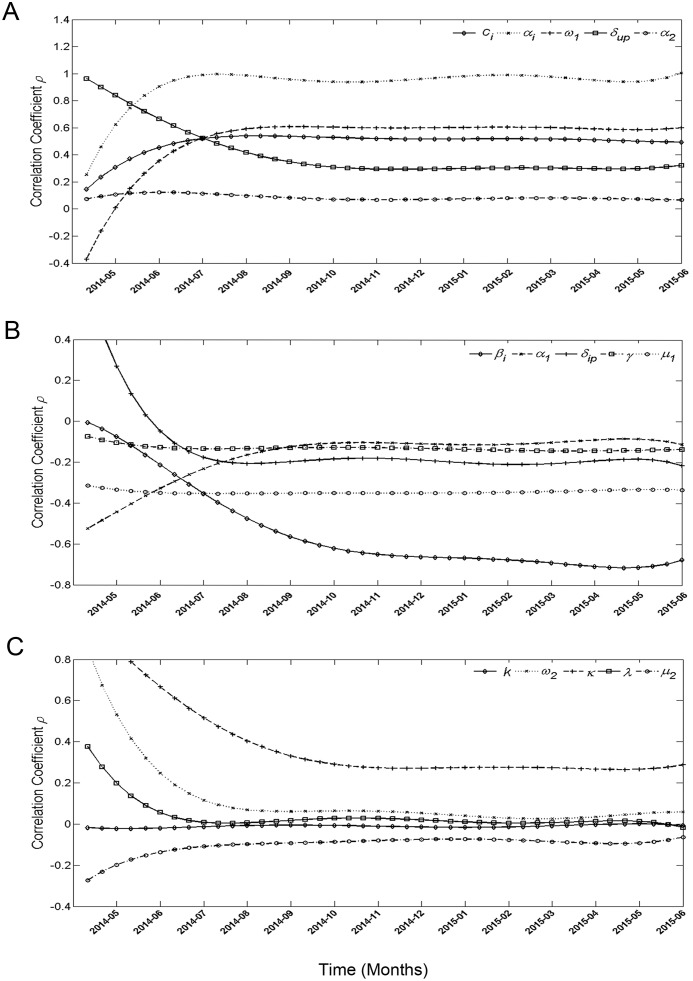
Sensitivity analysis for confirmed and suspected cases. (A) PRCCs that stay positive (>0) most of the time. (B) PRCCs that stay negative (<0) most of the time. (C) PRCCs that tend to stay near 0.

The sensitivity analysis was conducted with 15 parameters and a continuous time-series for confirmed and suspected cases. We took 1000 samples from a uniform distribution of each parameter range of the comprehensive compartment model. Partial rank correlation coefficients (PRCCs) of the parameters near -1 or +1 indicate a strong impact on the output. Parameters *α*_*i*_ and *β*_*i*_ had particularly high PRCCs (Fig 5), which means that the output of this model is highly sensitive to changes in *β*_*I*_.

## Discussion

An accurate analysis of the transmission pattern of the 2014 EVD epidemic provides a basis for implementing control and prevention measures. In this study, we constructed a dynamic transmission model to predict the epidemic trend of EVD and evaluated the effects of control and prevention measures. The key parameter in the model was the infection rate for patients in the free environment (*β*_*I*_). This parameter was influenced by a patient’s sphere of activity, the virulence of the virus, and the proportion of susceptible people in the total population. Fluctuations in *β*_*I*_ may cause the results of the model to vary. Thus, it is important to fit an infection rate that approximates real values closely. Here, we found that the distribution of *β*_*I*_ was approximated by a gamma distribution, rather than by an exponential distribution as an earlier study reported [22]. This discrepancy may be due to differences in the nature of the EVD epidemics modeled. In the 2014 epidemic, most of the early EVD cases were located in the sparsely populated rural areas, and the patients had a limited sphere of activity. When the disease spread to urban areas, patients came into contact with more susceptible individuals, causing the *β*_*I*_ value to increase. Subsequently, isolation measures for EVD cases and improvements in public efforts at self-protection reduced the number of close contacts between EVD patients and the susceptible population, ultimately reducing the *β*_*I*_ value.

Vaccination is an important means of preventing infectious disease [23]. Although there are no licensed EVD vaccines, we can estimate the demand for a vaccine, which may contribute to the formation of a vaccine and immunization program development. We estimated minimum effective vaccination rates for each country, and found that EVD would be controlled if greater than 42%, 45%, and 51% of the total population had immunity against the Ebola virus in Guinea, Liberia, and Sierra Leone, respectively. These results provide scientific evidence for vaccine production and application.

Assuming that no control and prevention measures were taken, understanding the effects of an unchecked EVD outbreak on the total population number would help us better appreciate how harmful EVD has the potential to be. Using an SIR model, we found that the population would be dramatically decreased in a short time in the absence of control and prevention measures. These results support an overwhelming risk from an EVD outbreak. The SIR model also indicated that the *s*, *i*, and *r* populations fluctuate periodically around their dynamic equilibrium points. These results support the idea EVD epidemics may break out periodically, though the disease will reach a dynamic equilibrium point with a shorter period between epidemics of progressively weaker intensity. Periodic fluctuations of EVD epidemics are similar to those of other diseases, such as the measles and chickenpox [24, 25]. Therefore, we should be vigilant for the reemergence of EVD.

The dynamic model constructed for the 2014–2015 EVD outbreak indicated that early detection, diagnosis, and isolation are critical for controlling EVD outbreaks. From March 2014 through January 2015, the mean time interval between symptom onset and sample collection from suspected patients was 7.2 days. The interval from sample collection to laboratory receipt was 1.5 days, 1.8 days, and 2.1 days in Guinea, Liberia, and Sierra Leone, respectively [26]. These relatively long intervals from symptom onset to diagnosis indicate that early detection of EVD is hindered by technical and social factors [26]. These delays likely contributed to the early expansion of the epidemic. Reducing them should improve transmission interruption, contact tracing, epidemiological surveillance accuracy, and prognoses.

Taking timely measures to control and prevent infection during small outbreaks is important for maintaining local control and preventing larger outbreaks. Previous studies have shown that strict isolation and quarantine, scientific management of corpses, public health education knowledge, and good personal hygiene habits are effective means of controlling and preventing the perpetuation of infectious diseases [27, 28]. Similarly, we found that the outbreak and spread of EVD could be controlled effectively through isolating patients early, quickly removing corpses, practicing “safe” sex after recovery, and strictly monitoring for imported cases. EVD patients were first reported in Guinea and then rapidly spread to neighboring countries that failed to prevent and manage imported cases effectively [1]. Preventing imported cases is a major way that countries can protect themselves from infectious disease. However, if imported cases do reach a country, an epidemic can still be prevented by rapidly implementing control and prevention measures. For example, several countries, including the USA and Spain, had imported EVD cases [29], but outbreaks were controlled successfully due to the timely isolation of cases and careful monitoring of close patient contacts.

One of the limitations of this study is that the epidemic data used were published by the WHO and were likely underreported due to the outdated public health system in West African countries. However, the WHO data are the most accurate data available at present. The constructed model fit and forecast epidemic trends accurately, evaluated the effects of control and prevention measures, and provided a scientific conclusion. In the SEIR model constructed during the 1995 EVD outbreak, the values of the model’s parameters were estimated based on stochastic process theory. However, in this study, the values of the model’s parameters were determined based on references or were simulated using real data from the 2014–2015 EVD outbreak. Therefore, the results of this study would be expected to more reliable because the model conformed more closely to the real-world circumstances.

In summary, this study indicated that EVD would be expected to re-emerge within two decades if control and prevention measures are not taken. To avoid similar EVD-related public health emergencies in the future, it is crucial that EVD vaccines are developed and approved for distribution in mass vaccination campaigns in Ebola virus-endemic areas. When future EVD cases are detected, it will be important for healthcare personnel to (1) isolate affected patients early, (2) trace and isolate infected patients’ close contacts quickly, (3) remove corpses with minimal delay, (4) conduct strict monitoring for imported cases, and (5) impose on recovered patients the importance of having condom-protected sex.

## Methods

### Estimation of the effective vaccination rate

The basic reproduction number (*R*_0_) was used to estimate the effective vaccination rate in the whole population. *R*_0_ represents the average number of secondary cases caused by an infected individual throughout the course of infection in a completely susceptible population and in the absence of control interventions. When *R*_0_<1, the disease epidemic will decline; when *R*_0_>1, disease spread will increase [30]. When random and continuous vaccination strategies for susceptible people were undertaken, the number of infected persons in the next generation will be less than 1 if at least *R*_0_−1 infected persons of the next generation were vaccinated, resulting in a termination of a pandemic. Therefore, the minimum vaccination rate of the population can be expressed as follow:

$$p_{v}=\frac{R_{0}-1}{R_{0}}=1-\frac{1}{R_{0}}$$

A detailed derivation of the above formula is published elsewhere [31].

### Epidemic trend without control measures

The epidemiology of EVD in the absence of control and prevention measures was analyzed by a SIR model. The differential equations were as follows:

$$\begin{array}{l} S\prime =bN-dS-\beta IS/N\\ I\prime =\beta IS/N-(\alpha +d+\gamma )I\\ R\prime =\gamma I-dR\\ N\prime =(b-d)N-\alpha I \end{array}$$ (1)

Because the birth rate (*b*) is greater than mortality rate (*d*) in West Africa, the population in this region is expected to increase and the number of individuals in the *S*, *I*, and *R* blocks should increase as well. Therefore, there was no dynamic equilibrium point in the system. The following normalization transformation was applied:

s=S/N, i=I/N, r=R/N

where

s+i+r≡1

The first three equations of equation group Eq (1) can be transformed as follows:

$$\begin{array}{l} s\prime =b-bs-\beta is+\alpha is\\ i\prime =\beta is-(\alpha +b+\gamma )i+\alpha i^{2}\\ r\prime =\gamma i-br+\alpha ir \end{array}$$ (2)

The expression of the modified reproductive number (designated by *θ*) was:

$$\theta =\frac{\beta }{\left(\alpha +b+\gamma \right)}$$

If *θ*≤1, then it can be proved that *s*, *i*, and *r* have a single dynamic equilibrium point of *P*_0_(1,0,0), and the system is asymptotically stable. If *θ*>1, then *P*_0_ is not stable, there is a single positive dynamic equilibrium point of P*(s*,i*,r*), and the system is asymptotically stable [32]. The calculation result (*θ* = 1.85), when the three countries are pooled into a single region, indicates the presence of a positive dynamic equilibrium point.

Next, the net growth threshold of the population was considered to analyze the trend of the total population *N* when *b*>*d* and *θ*>1. The net growth threshold of the population (designated by *φ*) can be expressed as:

$$\varphi =\frac{(b-d)\sigma }{\alpha (\sigma -1)}(1+\frac{\gamma }{d})$$

where

$$\sigma =\frac{\beta }{\left(\alpha +d+\gamma \right)}$$

If *φ*>1 and *t* → ∞, then *N*(*t*) → ∞; if *φ* = 1, then *N*(*t*) →N* (finite number); and if *φ<*1, then *N*(*t*) → 0 [32]. We estimated the epidemic trend of *s*, *i*, and *r* at the positive dynamic equilibrium point by applying the Jacobian matrix of equation group Eq (2) and then calculating the eigenvalue and feature vector. If the eigenvalue is a complex number, then the epidemic is in periodical oscillation.

### Evaluation of the control and prevention measures in the EVD outbreak

**Fig 6:**
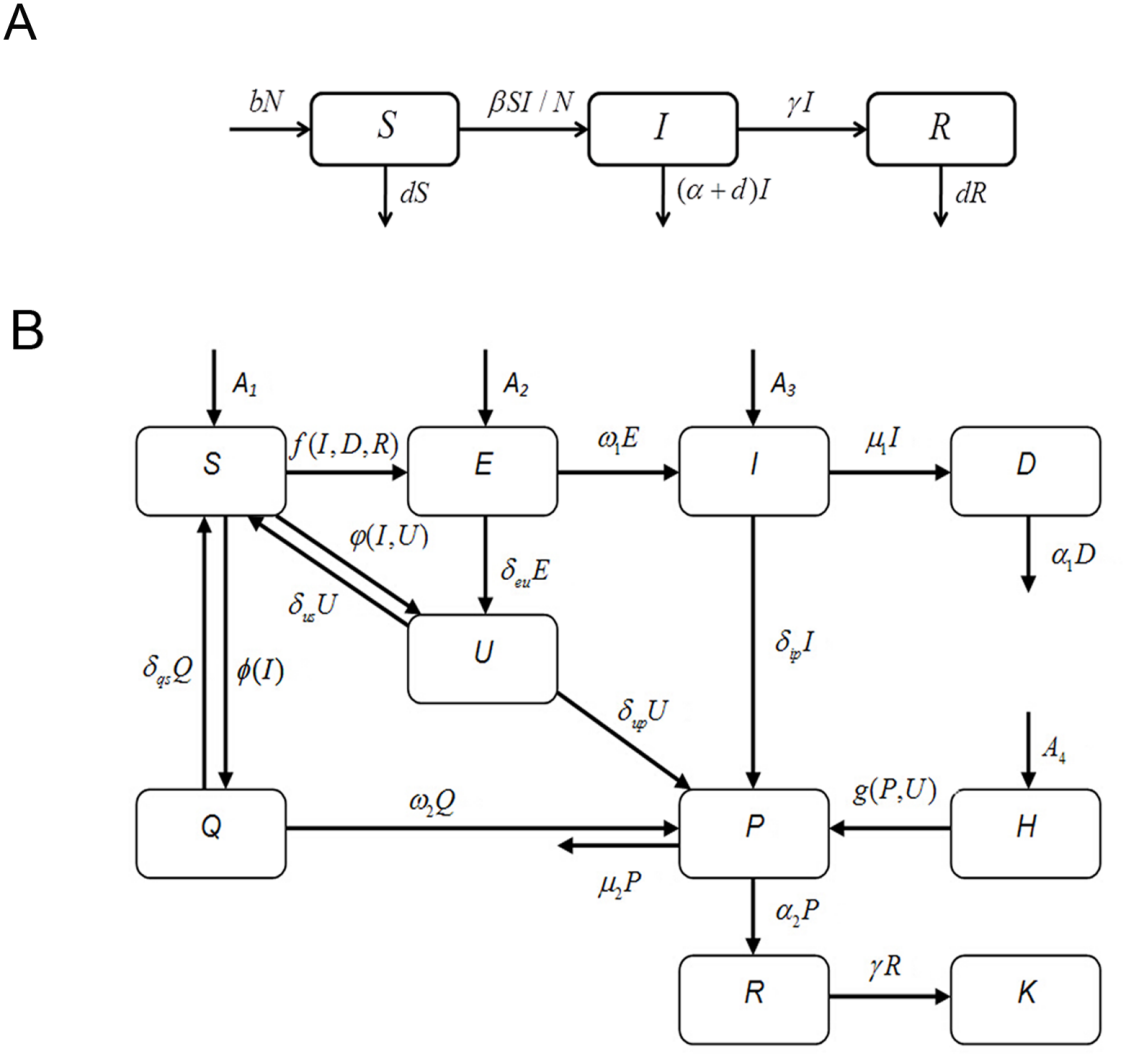
Compartment transfer block diagram of the transmission dynamic model. (A) Compartment transfer block diagram of the SIR model. Blocks *S*, *I*, and *R* represent susceptible individuals, symptomatic patients, and people with immunity owing to recovery, respectively. *β* is the standard contact rate, indicating the number of people infected by the same patient per unit time in a population of entirely susceptible persons. *α* and *γ* denote the probability of one patient dying or recovering per unit time, respectively. *b* and *d* are the population’s birth and mortality rates per unit time, respectively. (B) Comprehensive compartment transfer block diagram of EVD epidemiology. Blocks *S*, *E*, *I*, and *D* represent susceptible persons in a free environment, infected individuals in the incubation period, patients in a free environment, and not-yet-decontaminated corpses of EVD patients, respectively. Blocks *U*, *P*, and *Q* denote suspected cases in isolation, confirmed cases in isolation, and close contacts in isolation, respectively. Blocks *H*, *R*, *K*, and *A* indicate the medical staff in charge of *U* and *P*, recovered patients that are still infectious, recovered individuals that are not infectious and with immunity, and imported cases per day, respectively.

A compartment transfer block diagram was constructed based on the transmission patterns and control measures of EVD (Fig 6B). When a susceptible person in a free environment (*S*) comes in contact with a patient in a free environment (*I*), an un-decontaminated corpse of an EVD patient (*D*), or a recovering but still infectious patient (*R*), some people in *S* status become infected and enter the incubation period (*E*). People in *E* status can transfer to *I* or *U* status (suspected cases) to accept treatment and be isolated. Some people in the *S* block who are not infected, but present with EVD-like symptoms are transferred to the *U* block. Once a diagnosis is confirmed in suspected cases in the *U* block, these confirmed cases and some patients in the *I* block are both transferred to the *P* block (confirmed cases in isolation). Medical staff (*H*) are at high risk of infection due to close contact with people in the *U* and *P* blocks. Once an individual is confirmed to be in the *I* block, their close contacts are immediately monitored and isolated for observation (*Q* block). Some infected cases in the *Q* block are transferred to the *P* block after diagnosis. Uninfected cases return to the *S* status after the maximum incubation period. After treatment, some patients in the *P* block die and their corpses are rendered harmless by decontamination; however, other patients in the *P* block are transferred to the *R* block (recovered patients that are still infectious).

Differential equations were built according to the block diagram, as follows:

$$S\prime =A_{1}+\delta_{qs}Q+\delta_{us}U-f(I,D,R)-\varphi (I,U)-\phi (I)$$

$$E\prime =A_{2}+f(I,D,R)-\omega_{1}E-\delta_{eu}E$$

$$I\prime =A_{3}+\omega_{1}E-\mu_{1}I-\delta_{ip}I$$

$$D\prime =\mu_{1}I-\alpha_{1}D$$

$$U\prime =\delta_{eu}E+\varphi (I,U)-\delta_{us}U-\delta_{up}U$$

$$P\prime =\delta_{ip}I+\delta_{up}U+\omega_{2}Q+g(P,U)-\alpha_{2}P-\mu_{2}P$$

$$H\prime =A_{4}-g(P,U)$$

$$Q\prime =\phi (I)-\delta_{qs}Q-\omega_{2}Q$$

$$R\prime =\alpha_{2}P-\gamma R$$

K′=γR

where

$$f(I,D,R)=\beta_{I}(1-\delta_{i}{}_{p})I+\beta_{D}D+\beta_{R}R$$

$$\varphi (I,U)=\kappa (\delta_{ip}I+\delta_{up}U)$$

$$\phi (I)=1/k\beta_{I}\delta_{ip}I$$

$$g(P,U)=\beta_{P}(P+\lambda U)$$

After a confirmed case is identified in a free environment, his/her close contacts are checked within 24 hours and placed under observation in isolation. Therefore, the term *δ*_*ip*_*β*_*I*_*I* is subtracted from *f*(*I*,*D*,*R*). *κ* denotes the number of new suspected cases (non-EVD infection) when a new confirmed case is reported (excluding confirmed cases derived from the medical staff and close contacts). *λ* denotes the proportion of confirmed cases in the *U* block population. *k* represents the probability of a susceptible person being infected through close contact with a patient with EVD. All of these analyses were conducted in Matlab R2012a (MathWorks, USA, 2012).

The values of the parameters in the models shown in Fig 6A and 6B were obtained in three ways. The first way involved consultation of references, and the official websites of the WHO and the United Nations (noted as Reference in Table 1). For parameters that could not be obtained directly from published data, the second way, namely establishing the best fit model was used. The nonlinear least-squares method was used to achieve optimized curve-fitting in Matlab software. The values of parameters could be determined when the residual sum of squares of the simulation value and the WHO reported value was minimized (noted as nonlinear least- squares in Table 1). The third way was simple arithmetic calculations based on the values of parameters obtained from the former two approaches (noted as calculation in Table 1).

**Table 1:** Values, ranges and data sources of all parameters in models in Fig 6A and 6B.

Parameters	Values and ranges	Data sources
*R*_0_(Guinea)	1.710(1.440–2.010)	[3]
*R*_0_(Liberia)	1.830(1.720–1.940)	[3]
*R*_0_(Sierra Leone)	2.020(1.790–2.260)	[3]
*α*(Guinea)	0.110(0.104–0.116)	[3]
*α*(Liberia)	0.092(0.087–0.095)	[3]
*α* (Sierra Leone)	0.080(0.075–0.085)	[3]
*α*(All of them)	0.094(0.091–0.097)	[3]
*γ*(Guinea)	0.018(0.158–0.204)	[3]
*γ*(Liberia)	0.018(0.160–0.202)	[3]
*γ*(Sierra Leone)	0.018(0.156–0.021)	[3]
*γ*(All of them)	0.018(0.017–0.019)	[3]
*ω*(Guinea)	0.092(0.074–0.110)	[3]
*ω*(Liberia)	0.086(0.069–0.103)	[3]
*ω*(Sierra Leone)	0.093(0.074–0.112)	[3]
*ω*(All of them)	0.088(0.070–0.106)	[3]
*b*(Guinea)	1.093×10^−4^	[33, 34]
*b* (Liberia)	1.110×10^−4^	[33, 34]
*b* (Sierra Leone)	1.112×10^−4^	[33, 34]
*b*(All of them)	1.096×10^−4^	Calculation
*d*(Guinea)	3.808×10^−5^	[33, 34]
*d*(Liberia)	3.288×10^−5^	[33, 34]
*d*(Sierra Leone)	4.630×10^−5^	[33, 34]
*d*(All of them)	3.940×10^−5^	Calculation
*β*(Guinea)	0.220(0.185–0.259)	Calculation
*β* (Liberia)	0.201(0.189–0.213)	Calculation
*β*(Sierra Leone)	0.199(0.176–0.222)	Calculation
*β*(All of them)	0.210(0.187–0.234)	Calculation
*φ*(Guinea)	0.952	Calculation
*φ* (Liberia)	1.005	Calculation
*φ*(Sierra Leone)	0.901	Calculation
Population in Guinea	10 628 972	[35]
Population in Liberia	4 396 873	[35]
Population in Sierra Leone	6 190 280	[35]
*β*_*I*_	$c_{i}t^{\alpha_{i}}e^{-\beta_{i}t}$	Nonlinear least-squares
	*c*_*i*_:0.008(0.006–0.009)	
	*α*_*i*_:1.240(0.992–1.488)	
	*β*_*i*_:0.014(0.011–0.017)	
*β*_*D*_	$c_{d}+\alpha_{d}e^{-\beta_{d}t}$	Nonlinear least-squares
	*c*_*d*_:0.058(0.046–0.070)	
	*α*_*d*_:0.413(0.330–0.496)	
	*β*_*d*_:0.057(0.046–0.068)	
*β*_*R*_	$c_{r}+\alpha_{r}e^{-\beta_{r}t}$	Nonlinear least-squares
	*c*_*r*_:0.030(0.024–0.036)	
	*α*_*i*_:0.190(0.152–0.228)	
	*β*_*r*_:0.108(0.086–0.130)	
*κ*	1.200(0.960–1.440)	Nonlinear least-squares
*δ*_*qs*_	0.023(0.018–0.0276)	Calculation
*δ*_*us*_	0.047(0.038–0.056)	Calculation
*ω*_1_	0.035(0.026–0.044)	Nonlinear least-squares
*δ*_*eu*_	0.053(0.042–0.064)	Calculation
*μ*_1_	0.088(0.070–0.106)	Calculation
*δ*_*ip*_	0.054(0.043–0.065)	Calculation
*α*_1_	0.500(0.333–1.000)	Nonlinear least-squares
*δ*_*up*_	0.075(0.060–0.090)	Calculation
*ω*_2_	0.065(0.052–0.078)	Calculation
*β*_*P*_	$c_{p}+\alpha_{p}e^{-\beta_{p}t}$	Nonlinear least-squares
	*c*_*p*_:0.004(0.003–0.005)	
	*α*_*p*_:0.101(0.081–0.121)	
	*β*_*p*_:0.190(0.152–0.228)	
*α*_2_	0.031(0.025–0.037)	Calculation
*μ*_2_	0.155(0.124–0.186)	Calculation
*γ*	0.033(0.025–0.050)	Calculation
*k*	0.740(0.592–0.888)	Nonlinear least-squares
*λ*	0.460(0.368–0.552)	Nonlinear least-squares

### Sensitivity analysis of the comprehensive compartment

PRCC determination combined with Latin hypercube sampling (LHS) was used for the sensitivity analysis. The PRCC is an efficient and reliable sampling-based sensitivity analysis method that provides a measure of monotonicity between a set of parameters and the model output after removing the linear effects of all parameters except the parameter of interest [36]. LHS is a stratified Monte Carlo sampling method in which each parameter’s range is divided into *N* equal intervals and one sample is selected randomly from each interval [36, 37]. A standard correlation coefficient-*ρ* for the parameter and model output was calculated.

## Supporting Information

S1 Table The No. of cumulative cases and deaths published by WHO. (DOCX)
