# Supplementary material for: Predicting and Evaluating the Epidemic Trend of Ebola Virus Disease in the 2014-2015 Outbreak and the Effects of Intervention Measures
Source: PLoS One. 2016 Apr 6;11(4):e0152438. doi: 10.1371/journal.pone.0152438 (PMC4822846; doi:10.1371/journal.pone.0152438)
Supplement: S1 Table — (DOCX) [file pone.0152438.s001.docx]

**S1 Table. The No. of cumulative cases and deaths published by WHO**

| **Data** | **No. of cumulative confirmed and suspected cases** | **No. of cumulative deaths** |
| --- | --- | --- |
| \| 2014-04-01 \| \| --- \| \| 2014-04-04 \| \| 2014-04-11 \| \| 2014-04-15 \| \| 2014-04-17 \| \| 2014-04-24 \| \| 2014-04-29 \| \| 2014-05-08 \| \| 2014-05-17 \| \| 2014-05-21 \| \| 2014-05-27 \| \| 2014-05-30 \| \| 2014-06-04 \| \| 2014-06-12 \| \| 2014-06-18 \| \| 2014-06-26 \| \| 2014-07-02 \| \| 2014-07-04 \| \| 2014-07-09 \| \| 2014-07-12 \| \| 2014-07-14 \| \| 2014-07-17 \| \| 2014-07-21 \| \| 2014-07-24 \| \| 2014-07-26 \| \| 2014-07-29 \| \| 2014-07-31 \| \| 2014-08-03 \| \| 2014-08-05 \| \| 2014-08-07 \| \| 2014-08-10 \| \| 2014-08-12 \| \| 2014-08-14 \| \| 2014-08-20 \| \| 2014-08-25 \| \| 2014-08-28 \| \| 2014-09-01 \| \| 2014-09-04 \| \| 2014-09-08 \| \| 2014-09-11 \| \| 2014-09-15 \| \| 2014-09-17 \| \| 2014-09-19 \| \| 2014-09-22 \| \| 2014-09-25 \| \| 2014-09-29 \| \| 2014-10-01 \| \| 2014-10-06 \| \| 2014-10-11 \| \| 2014-10-13 \| \| 2014-10-18 \| \| 2014-10-24 \| \| 2014-10-27 \| \| 2014-10-29 \| \| 2014-11-03 \| \| 2014-11-05 \| \| 2014-11-10 \| \| 2014-11-12 \| \| 2014-11-17 \| \| 2014-11-24 \| \| 2014-12-01 \| \| 2014-12-08 \| \| 2014-12-15 \| \| 2014-12-22 \| \| 2014-12-29 \| \| 2015-01-05 \| \| 2015-01-12 \| \| 2015-01-19 \| \| 2015-01-26 \| | \| 163 \| \| --- \| \| 169 \| \| 209 \| \| 215 \| \| 220 \| \| 233 \| \| 243 \| \| 245 \| \| 270 \| \| 309 \| \| 354 \| \| 438 \| \| 474 \| \| 528 \| \| 599 \| \| 759 \| \| 844 \| \| 888 \| \| 964 \| \| 1048 \| \| 1093 \| \| 1201 \| \| 1323 \| \| 1440 \| \| 1603 \| \| 1711 \| \| 1779 \| \| 1848 \| \| 1975 \| \| 2127 \| \| 2240 \| \| 2473 \| \| 2615 \| \| 3069 \| \| 3707 \| \| 4001 \| \| 4366 \| \| 4846 \| \| 5339 \| \| 5762 \| \| 6263 \| \| 6574 \| \| 6808 \| \| 7192 \| \| 7492 \| \| 8033 \| \| 8386 \| \| 8997 \| \| 9693 \| \| 9964 \| \| 11868 \| \| 12647 \| \| 13041 \| \| 13268 \| \| 14098 \| \| 14413 \| \| 15145 \| \| 15351 \| \| 15935 \| \| 17145 \| \| 17942 \| \| 18603 \| \| 19497 \| \| 20206 \| \| 20747 \| \| 21296 \| \| 21724 \| \| 22092 \| \| 22495 \| | \| 82 \| \| --- \| \| 88 \| \| 102 \| \| 108 \| \| 129 \| \| 136 \| \| 143 \| \| 153 \| \| 162 \| \| 164 \| \| 181 \| \| 200 \| \| 208 \| \| 231 \| \| 252 \| \| 337 \| \| 338 \| \| 467 \| \| 518 \| \| 539 \| \| 603 \| \| 632 \| \| 660 \| \| 672 \| \| 729 \| \| 826 \| \| 887 \| \| 932 \| \| 961 \| \| 1013 \| \| 1069 \| \| 1145 \| \| 1229 \| \| 1350 \| \| 1427 \| \| 1552 \| \| 1848 \| \| 2089 \| \| 2177 \| \| 2375 \| \| 2586 \| \| 2746 \| \| 2900 \| \| 3043 \| \| 3159 \| \| 3286 \| \| 3439 \| \| 3865 \| \| 3988 \| \| 4485 \| \| 4811 \| \| 4877 \| \| 4960 \| \| 5026 \| \| 5087 \| \| 5160 \| \| 5177 \| \| 5191 \| \| 5420 \| \| 5459 \| \| 5689 \| \| 6070 \| \| 6388 \| \| 6915 \| \| 7588 \| \| 7905 \| \| 8235 \| \| 8429 \| \| 8641 \| |
